# Assemblages of certain benthic molluscs along the southwestern Atlantic: from subtidal to deep sea

**DOI:** 10.1186/s12898-019-0263-7

**Published:** 2019-11-27

**Authors:** Valeria Teso, Diego Urteaga, Guido Pastorino

**Affiliations:** Laboratorio de Ecosistemas Costeros, Plataforma y Mar Profundo, Museo Argentino de Ciencias Naturales “Bernardino Rivadavia”, Av. Angel Gallardo 470, C1405DJR Ciudad de Buenos Aires, Argentina

**Keywords:** Biogeography, MPA Namuncurá/Burdwood Bank, Continental shelf, Mar del Plata Submarine Canyon

## Abstract

**Background:**

We analyse the distribution of Gastropods and Chitons from shallow to deep waters along the southwestern Atlantic Ocean off Argentina and discuss possible factors determining the observed biogeographic patterns.

**Results:**

Three major biogeographic groups are defined on the basis of Gastropod and Chiton species associations, i.e., continental shelf (< 350 m), upper continental slope (> 350–2000 m) and lower continental slope (2000–3000 m). Bathymetry appears as the main factor modifying large-scale distribution of the fauna at a. In this scenario, species associations are determined by marine currents that clearly occur at a particular and well defined depth. No arrangement of species by geographic location was found in assemblages from the continental shelf and upper continental slope.

**Conclusions:**

We hypothesize that depth and marine currents are the main factor affecting the distribution of Gastropods and Chitons along the SW Atlantic between 200 and 3000 m depth.

## Background

Over the past two decades several works have reported patterns of biogeography of benthic communities from diverse regions around the world. These patterns have been attributed to different factors e.g., depth, type of substratum, salinity, latitude, shelf area, food supply, etc. [8, 12, 14–17, 30, 38; among others]. Many of these studies were focused on molluscs, probably because they constitute one of the most abundant and diverse groups in the marine realm and were used successfully to explain general biogeographic patterns of benthic invertebrates as a whole [1, 6, 10, 32, 36, 37].

The diversity of marine currents of different geographical provenances in a certain area is important to understand the distribution and origins of the recent fauna. In this sense, the southwestern Atlantic at ~ 38° S is strongly influenced by the Malvinas current (MC), Brazil current (BC) and the Brazil-Malvinas Confluence (BMC) on the continental shelf [20, 27, 40; among others]. Other currents affect the continental slope depending on depth. In this way, Piola and Matano [26], Violante et al. [39] and Voigt et al. [40] among others, recognized the Antarctic Intermediate Water (AAIW) at ~ 500 to 1000 m depth; the Upper Circumpolar Deep Water (UCDW) at ~ 1000 to 2000 m; the North Atlantic Deep Water (NADW) at 2000 to 3000 m; the Lower Circumpolar Deep Water (LCDW) at 3000 to 4000 m and the Antarctic Bottom Water (AABW) at > 4000 m depth. The southwestern Atlantic shelf extends from Cape Frio, Brazil (~ 22° S) to Tierra del Fuego and Burdwood Bank, Argentina (~ 55° S). It is the largest continental shelf in the southern hemisphere and one of the most energetic oceanic regions of the world [20, 26].

Ecological studies of benthic marine communities off Argentina were mostly based on faunas from shallow water areas. Benthic shallow-water communities off Mar del Plata (~ 38° S) in Buenos Aires province are one of the better studied zones in this country [21, 29, 42]. General approaches on the Argentine continental shelf at large were seldom taken. Nevertheless, Bastida et al. [7] reported benthic macroinvertebrate assemblages of this area up to 192 m depth and Balech and Ehrlich [4] studied the fauna of the Argentine shelf stressing the already known Argentine and Magellanic biogeographic provinces. Undoubtedly because shallow-water areas are easier to sample, biodiversity and biogeography of these areas are better known than deep-water ones (a list with all these references is available in Additional file 1).

In contrast, publications addressing the ecology of deeper benthic communities off Argentina are scarce. Most papers are reports of diversity and species richness of several groups of invertebrates. Those made by Riemann-Zürneck [28] (Cnidarians); Allen and Sanders [3] (Bivalves); López-Gappa [18] (Bryozoans); Zamponi [41]; Linse et al. [17] (Molluscs); López-Gappa, et al. [19] (Amphipods); Barnes and Griffiths [5] and Allen [2] (bivalves) establish the starting point of deep-waters studies in this area. In addition, Stuart and Rex [35] reported some bathymetric patterns of deep-sea Gastropods in the vicinity of the Rio de la Plata estuary off Uruguay and Argentina. Bremec and Schejter [9] mentioned benthic diversity from ~ 43° S, 59° W at ~ 300 m depth and Fraysse et al. [13] reported the geographic and bathymetric distribution of asteroidean echinoderms off Tierra del Fuego and Burdwood Bank at 785 m depth.

As molluscs are one of the most abundant and better known groups of benthic invertebrates, in this study we analyse the distribution of the Gastropods and chitons from shallow to deep water (up to 3000 m) along the southwestern Atlantic Ocean off Argentina and discuss possible factors determining the observed biogeographic patterns.

## Methods

**Fig. 1:**
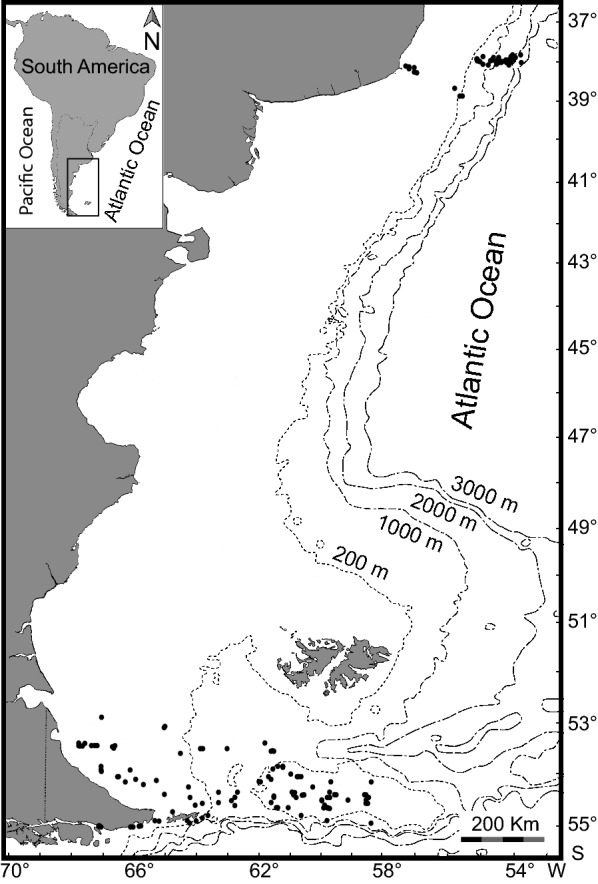
Map showing all sampling localities, dashed lines represent isobates

Three areas off Argentina were covered: (1) along 38° S, from ~ 40 m to 2900 m depth; (2) Burdwood Bank and vicinity at ~ 80 and 1000 m depth; (3) off Tierra del Fuego between ~ 40 and 330 m depth (Fig. 1). Invertebrates in general and molluscs in particular were sampled on board of the R/V “Puerto Deseado” from 10 stations off Buenos Aires Province during October 2009; 41 stations in the Mar del Plata Submarine Canyon on August 2012, May and September 2013; 48 stations off Tierra del Fuego in March 2011, March–April 2014, November 2014, March–April 2016 and May 2017; and 64 stations in the Burdwood Bank area in March 2013, November 2014, March–April 2016 and May 2017. All material was collected using three different sampling gear: bottom net trawl, modified Agassiz dredge and Rauschert dredge, then preserved in ethanol 96%. Special care was taken to include only those species collected alive and with taxonomic status resolved. According with these restrictions we agree with Scarabino et al. [32], who suggested that inferred ranges of geographic or bathymetric distribution of shelly marine invertebrates are distorted by unresolved/non-reviewed taxonomy and, living/dead status not specified in records, among several other reasons.

The database was built by determining the presence/absence of species in the sampled area. All the species were arranged in seven sections defined by the marine currents reported by Matano et al. [20], Preu et al. [27], Voigt et al. [40] and Pérez et al. [25]. These regions are: Mar del Plata Continental Shelf (MCS) (38–300 m), Mar del Plata Submarine Canyon (MSC) (300–1000 m), (1000–2000 m), (2000–3000 m); Burdwood Bank (BB) (80–200 m); Burdwood Bank Continental Shelf and Slope (BBCSS) (200–1000 m) and off Tierra del Fuego (TF) (40–324 m). Hierarchical agglomerative clustering was undertaken using group-average link on Sorensøn association coefficients calculated from a presence/absence species matrix [10]. A non-metric multidimensional scaling (NMDS) was then performed using the similarity matrix. Multivariate analyses were completed using PRIMER v6.0 software [11].

## Results

**Fig. 2:**
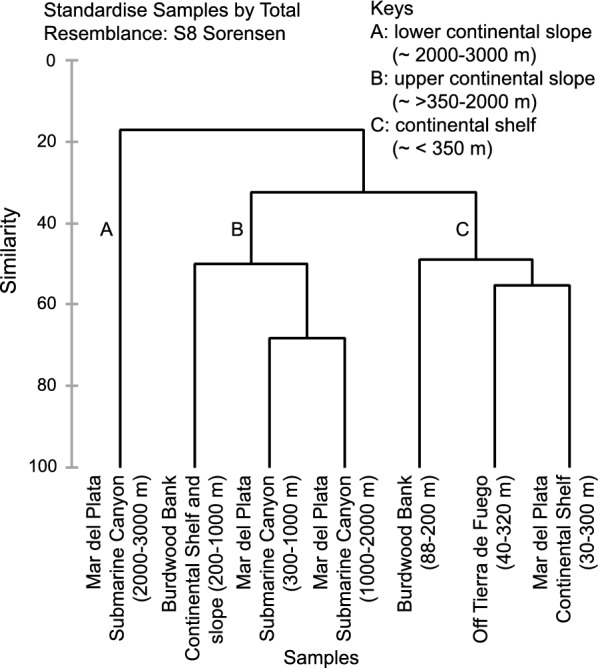
Hierarchical agglomerative clustering of sections based on presence/absence matrix of Gastropods and Chitons with depths

**Fig. 3:**
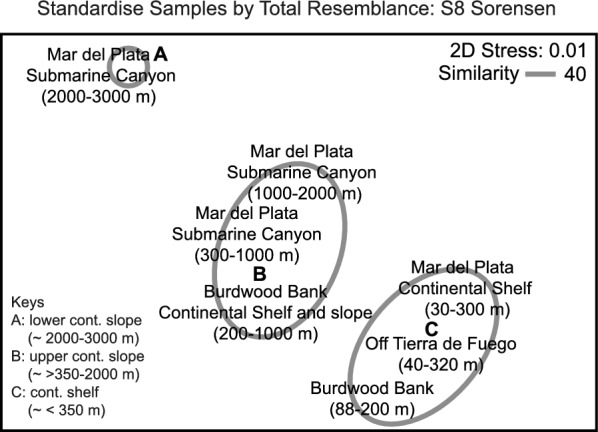
Non-metric multidimensional scaling of sections, stress value = 0.01

A total of 52 species of 19 families of Gastropods and Chitons were found and included in this study in 163 stations (dataset available in Additional file 2). Hierarchical agglomerative clustering of areas according to marine currents based on presence/absence matrix separates three groups corresponding to continental shelf (< 350 m) (Group C), upper continental slope (> 350–2000 m) (Group B) and lower continental slope (2000–3000 m) (Group A) (Fig. 2). As expected, the groups of the NMDS resembled those identified by clustering with a significant stress value of 0.01 (Fig. 3).

**Fig. 4:**
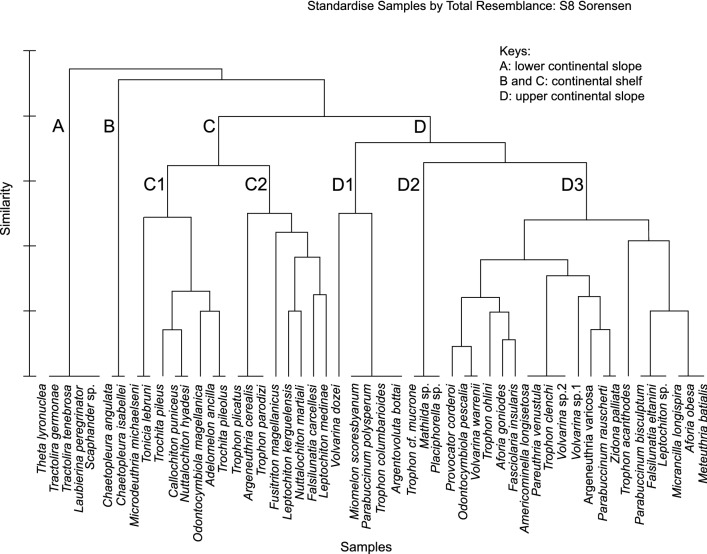
Hierarchical agglomerative clustering of 53 species of Gastropods and Chitons based on presence/absence matrix in the sampled area

Cluster analysis of species separates four groups and the NMDS ordination (Fig. 4) showed similar results to those of the dendrogram, with a significant stress value of 0.08. The Group A was represented by the Gastropods *Laubierina peregrinator* Warén & Bouchet, 1990, *Tractolira germonae* Harasewych, 1987, *Tractolira tenebrosa* Leal & Bouchet, 1985, *Theta lyronuclea* (A. H. Clarke, 1959) and *Scaphander* sp. and were exclusive of the lower continental slope. The Group B and C belong to the continental shelf, and Group B was integrated only by *Chaetopleura angulata* (Spengler, 1797) and *Chaetopleura isabellei* (d’Orbigny, 1841), two chitons, which were exclusive to the MCS area. Cluster D was similar to the upper continental slope area and was subdivided in three subgroups: D1 including species aggregated in BBCSS (200–1000 m) and one species, *Volvarina dozei* (Mabille & Rochebrune, 1889), found in all areas except BB; D2 species of MSC (1000–2000 m) and D3 species of the upper continental slope areas. Group C and D appear closer to each other than to Group A.

**Fig. 5:**
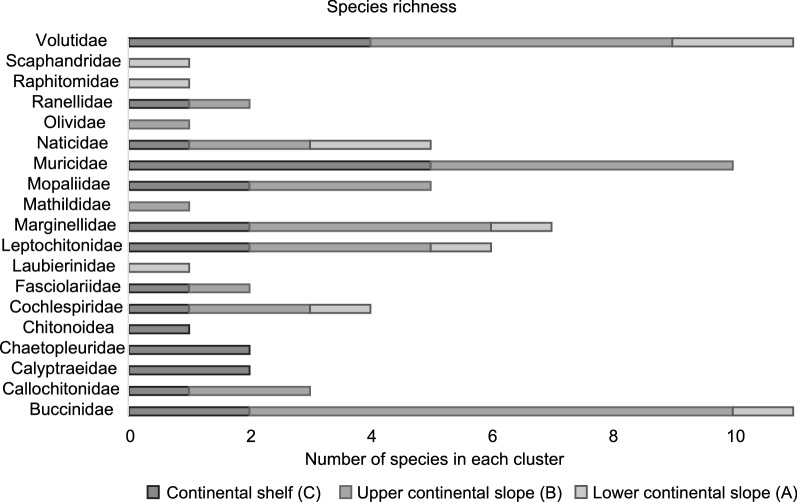
Species richness of families of Gastropods and Chitons analysed

The most diverse families were the Gastropods: Volutids with nine species, Buccinids and Muricids with eight species each one. Species richness in each group is shown in Fig. 5. In Cluster A all families were represented in similar numbers except for Volutidae and Naticidae that predominated. In Cluster C Muricidae and Volutidae were the most abundant, while in Cluster B, Buccinidae, Muricidae and Volutidae are in majority.

## Discussion

Gastropod and chiton species composition defined three major biogeographic groups, i.e., continental shelf (< 350 m), upper continental slope (> 350–2000 m) and lower continental slope (2000–3000 m). Within the upper continental slope group, samples from MSC (350–2000 m) grouper together, due to a probably secondary geomorphological factor. According to the former grouping by depth Troncoso et al. [37] described a bathymetric pattern from deep-water stations (depth > 438 m) to shallow water ones (depth < 410 m) in the Antarctic Peninsula. Later, Aldea et al. [1] reported three bathymetric boundaries in the same area up to 2000 m depth and Saiz et al. [30] found a depth gradient from deeper stations of the western sector of the Bellingshausen sea to the eastern shallow water stations closer to the Antarctic Peninsula in benthic assemblages. In addition, Carranza et al. [10] reported two major groups of megabenthic Gastropods in the inner and outer shelves up to 850 m depth in the Uruguayan and northern Argentine shelves.

The causes of bathymetric zonation are complex, and several biological and physical factors may act together to produce the obtained patterns. The studied area is dominated by the action of intermediate and deep-water currents, including—from top to bottom—AAIW, UCDW, NADW, LCDW and AABW [26, 39, 40; among others]. All these water masses have different depths, densities, salinities and temperatures, and transport diverse sediments that could be responsible for the macrobenthic assemblages. Carranza et al. [10] suggested dissimilar tolerances to salinity and depth and different water masses as possible factors determining variations between Gastropod assemblages in the inner and outer shelf off Uruguay. In a different approach Troncoso et al. [37] considered that depth and percentage of coarse sand could be the environmental variables that explain the distribution of molluscan clusters in the Bellingshausen sea. Allen [2] reported food supply and high pressure on the physiology of body functions as the main environmental factors that control the occurrence and evolution of the bivalves in the deep-sea Atlantic.

Several macrobenthic assemblages of shallow and intermediate waters were associated by several authors with ecoregions or biogeographic provinces. Linse et al. [17] defined three biogeographic provinces of molluscan fauna in the southern ocean up to 1000 m depth. In addition, Barroso et al. [6] reported four regions working with prosobranch Gastropods from Brazil down to 200 m depth. In agreement with these previous authors, Hernández-Âvila et al. [15] also detected changes in species composition in relation to ecoregions and depth in the Caribbean.

In this work, species seem to be arranged by the characteristics of the water mass they inhabit, instead of by their geographic position as proposed by Linse et al. [17], Barroso et al. [6] and Hernández-Âvila et al. [15; among others]. However, this is not applicable to Group A because of the lack of samples between 2000 and 3000 m depth at high latitudes. Group A is composed by species living only below 2000 m depth in the Mar del Plata Submarine Canyon area. *Laubierina peregrinator*, *Theta lyronuclea* and *Scaphander* sp. were recently reported in this area [23, 31, 34]. While the particular larval biology of tonnoideans as *L. peregrinator*, with teleplanic larvae could explain the extraordinary distribution of some of these species, others have no clear explanation. The term teleplanic was coined by Scheltema [33] and followed by several authors after studying larvae from different unrelated groups as Gastropods, Echinoderms or Crustaceans among others. It refers to those larvae that delay settlement and have adaptations for a long planktonic life rendering thus a possible widespread geographic distribution.

Species Group C (38–350) and D (350–2000 m) are defined by a larger bathymetric range than species Group A. Furthermore, certain overlap of species involving C and D is recorded (see Additional file 2). In this way, some proximity between C and D is to be expected.

Within Cluster C1 there are two species that were grouped and collected exclusively in the BB i.e.: *Microdeuthria michaelseni* (Strebel, 1905) and *Tonicia lebruni* Rochebrune, 1884. However, *M. michaelseni* was recorded off Buenos Aires Province and Tierra del Fuego between 56 and 293 m [24]. In Group C2 there are three species that were found exclusively in TF, however *Trophon plicatus* (Lightfoot, 1786) was reported from Peninsula Valdés to TF [22] and *Argeneuthria cerealis* (Rochebrune & Mabille, 1885) from Puerto San Julián, Santa Cruz to TF [24]. According to our data there are no endemic species from the Burdwood Bank; yet, studies in the intermediate regions between ~ 52° S and off Mar del Plata are necessary to confirm this.

The distribution of shallow-water molluscs studied in this work follows the traditional scheme of biogeographic provinces (i.e. Argentine and Magellanic) reported by previous authors [4, 7; among others]. Some exceptions are the Gastropods *Adelomelon ancilla* (Lightfoot, 1786) and *Trochita pileus* (Lamarck, 1822), which are fairly common in both provinces. Bastida et al. [7] described a third area—according to the sampling of benthic macroinvertebrates—extending along the Argentine continental shelf between 100 and 200 m depth. In our work, molluscs were grouped by bathymetry and species distributed between 100 and 200 m depth were clustered in Group C including those from the continental shelf (350 m). A possible explanation for the differences in this third area could be attributed to differences in sampling bathymetry, as Bastida et al. [7] only collected macroinvertebrates from shallow water areas up to 200 m depth instead of 3000 m.

## Conclusions

Our results showed that species were aggregated in agreement with marine currents occurring at a particular and well-defined depth with certain variability according to latitude. Thus, bathymetry appears as the main factor modifying large-scale distribution of the fauna. More samples of deep-sea species (i.e. 1000–3000 m) at 53ºS–56ºS are required to compare with the species in Group A. Furthermore, fieldwork needs to be carried out between 39º S and 53º S, following shallow and deep currents (i.e. MC, BC, AAIW, UCDW, NADW, LCDW, AABW) in order to assess the species distribution patterns between the northern and southern areas analysed herein, and eventually increase the knowledge of the factors that contribute to global-scale benthic patterns.

This is the first comparative report of biogeography of Gastropods and Chitons along the Argentine continental shelf including species up to ~ 3000 m depth.

## Supplementary information

**Additional file 1.** Description of data: Bibliographic references to publications referring to biodiversity and biogeography studies in shallow waters of southwestern Atlantic.

**Additional file 2**. Description of data: Stations where invertebrate material was sampled with latitude, longitude and depth.

## Data Availability

All data generated or analysed during this study are presented within the manuscript and/or additional supporting files.

## Abbreviations

AABW: Antarctic Bottom Water
AAIW: Antarctic Intermediate Water
BB: Burdwood Bank
BBCSS: Burdwood Bank Continental Shelf and Slope
BC: Brazil Current
BMC: Brazil-Malvinas Confluence
LCDW: Lower Circumpolar Deep Water
MC: Malvinas Current
MCS: Mar del Plata Continental Shelf
MSC: Mar del Plata Submarine Canyon
NADW: North Atlantic Deep Water
NMDS: non-metric multidimensional scaling
UCDW: Upper Circumpolar Deep Water
TF: off Tierra del Fuego
