# Supplementary material for: Assemblages of certain benthic molluscs along the southwestern Atlantic: from subtidal to deep sea
Source: BMC Ecol. 2019 Nov 27;19:49. doi: 10.1186/s12898-019-0263-7 (PMC6880383; doi:10.1186/s12898-019-0263-7)
Supplement: Supplementary file 1 — Additional file 1. Description of data: Bibliographic references to publications referring to biodiversity and biogeography studies in shallow waters of southwestern Atlantic. [file 12898_2019_263_MOESM1_ESM.docx]

*Additional file 1*

Arribas LP, Martínez MI, Brogger MI. Echinoderms in San Matías Gulf, Southwestern Atlantic Ocean. Thalass. 2006;32:11-8.

Bremec C, Brey T, Lasta M, Valero J, Lucifora L. *Zygochlamys patagonica* beds on the Argentinian shelf: Part I: energy flow thought the scallop bed community. Arch of Fish and Mar Res. 2000;48:295-303.

Bremec C, Giberto D. Polychaete assemblages in the Argentinean Biogeographical Province, between 34° and 38° S. Sci Mar. 2006;70:249-258.

Brogger MI, Gil DG, Rubilar T, Martinez MI, Díaz de Vivar E, Tablado A (2013) Echinoderms from Argentina: Biodiversity, Distribution and Current State of Knowledge. In Alvarado JJ, Solís-Marín FA, editors. Echinoderms Research and Diversity in Latin America. Berlin Heidelberg: Springer-Verlag; 2013. P. 359-402.

Doti BL, Roccatagliata D, López-Gappa J. An inverse latitudinal biodiversity pattern in asellote isopods (Crustacea, Peracarida) from the Southwest Atlantic between 35° and 56°S. Mar Biodivers. 2014;44:115-25.

Escolar M, Hernández DR, Bremec CS. Latitudinal and bathymetric distribution patterns of ophiuroids (Echinodermata: Ophiuroidea) on scallop fishing grounds at the shelf-break frontal system, south-western Atlantic. J of the Mar Biol Assoc of the U K. 2013;6:e137.

Schejter L, Rimondino C, Chiesa I, Díaz de Astarloa JM, Doti B, Elías, R, et al. Namuncurá Marine Protected Area: an oceanic hot spot of benthic biodiversity at Burdwood Bank, Argentina. Polar Biol. 2016;39:2373-86.

Souto V, Escolar M, Genzano G, Bremec C. Species richness and distribution patterns of echinoderms in the southwestern Atlantic Ocean (34-56°S) Sci Mar. 2014;78:269-280.

Zamponi MO, Acuña FH. Zoogeografía y algunos aspectos ecológicos de la fauna de anémonas de la provincia Magallánica. Neotróp. 1991;37:95-105.

Zamponi MO, Genzano GN, Acuña FG, Excoffon AC. Studies of Benthic Cnidarian Taxocenes along a Transect off Mar del Plata (Argentina). Russ J of Mar Biol. 1998;24:7-13.
